# Baseline systolic blood pressure, hypertension history, and efficacy of remote ischemic conditioning

**DOI:** 10.1002/acn3.52077

**Published:** 2024-06-03

**Authors:** Ji‐Ru Cai, Nan‐Nan Zhang, Yu Cui, Yue‐Xin Ning, Qiong Wu, Yi‐Na Zhang, Hui‐Sheng Chen

**Affiliations:** ^1^ Department of Neurology General Hospital of Northern Theater Command Shenyang China; ^2^ Department of Neurology Postgraduate Training Base of Jinzhou Medical University in the General Hospital of Northern Theater Command Shenyang China

## Abstract

**Objective:**

We performed a post hoc exploratory analysis of Remote Ischemic Conditioning for Acute Moderate Ischemic Stroke (RICAMIS) to determine whether hypertension history and baseline systolic blood pressure (SBP) affect the efficacy of remote ischemic conditioning (RIC).

**Methods:**

Based on the full analysis set of RICAMIS, patients were divided into hypertension versus non‐hypertension group, or <140 mmHg versus ≥140 mmHg group. Each group was further subdivided into RIC and control subgroups. The primary outcome was modified Rankin Scale (mRS) 0–1 at 90 days. Efficacy of RIC was compared among patients with hypertension versus nonhypertension history and SBP of <140 mmHg versus ≥140 mmHg. Furthermore, the interaction effect of treatment with hypertension and SBP was assessed.

**Results:**

Compared with control group, RIC produced a significantly higher proportion of patients with excellent functional outcome in the nonhypertension group (RIC vs. control: 65.7% vs. 57.0%, OR 1.45, 95% CI 1.06–1.98; *p* = 0.02), but no significant difference was observed in the hypertension group (RIC vs. control: 69.1% vs. 65.2%, *p* = 0.17). Similar results were observed in SBP ≥140 mmHg group (RIC vs. control: 68.0% vs. 61.2%, *p* = 0.009) and SBP <140 mmHg group (RIC vs. control: 65.6% vs. 64.7%, *p* = 0.77). No interaction effect of RIC on primary outcome was identified.

**Interpretation:**

Hypertension and baseline SBP did not affect the neuroprotective effect of RIC, but they were associated with higher probability of excellent functional outcome in patients with acute moderate ischemic stroke who received RIC treatment.

## Introduction

Reperfusion such as intravenous thrombolysis and endovascular treatment has been demonstrated as the best treatment for acute ischemic stroke (AIS), but only a small number of patients can receive these treatments due to the limited time window and the requirements of equipment and skilled operators.^1^, ^2^, ^3^ Given these limitations of reperfusion therapies, cerebroprotective treatment has been a hot research topic to improve clinical outcome in AIS. To date, no drugs have been successfully translated into clinical use, although a lot of drugs showed cerebroprotective effect in preclinical studies.^4^, ^5^ Since the phenomenon of myocardial ischemic preconditioning^6^, increasing evidence has suggested the potentially cerebroprotective effect of remote ischemic conditioning (RIC) on AIS in preclinical and clinical studies, which involved multiple mechanisms such as inhibiting neuroinflammation, anti‐oxidative stress, and improving cerebral circulation.^7^, ^8^, ^9^ There has been a lack of robust evidence for the neuroprotective effect of RIC in patients with AIS due to small sample sizes, different RIC procedures, and heterogeneity of patients, with varying extents of neurological deficits.^10^, ^11^, ^12^, ^13^, ^14^ In this context, we designed the RICAMIS trial and the results showed that 2‐week RIC significantly improved clinical outcome in acute moderate ischemic stroke, which further provide the evidence for the cerebroprotective effect of RIC on stroke.^15^

As the major risk factor for stroke, hypertension impaired cerebral autoregulation, which contributed to poor outcome after ischemic stroke.^16^, ^17^ In addition, too high or low systolic blood pressure (SBP) was also found to be related to impaired cerebral autoregulation^18^, ^19^ and poor clinical outcome after stroke.^20^, ^21^, ^22^ Interestingly, a recent study has shown that RIC has the potential to lower blood pressure.^23^ In this context, we hypothesize that hypertension and SBP may affect efficacy of RIC in ischemic stroke, given their effect of RIC on cerebral autoregulation.^24^ The issue is of importance but is never investigated. In this post hoc secondary analysis, we aimed to investigate this issue based on RICAMIS data.

## Methods

### Study design and participants

Details on the design, procedures, and statistical analysis plan for RICAMIS trial have been previously published^15^. In short, the RICAMIS study was a multicenter, open label, blinded‐endpoint, randomized clinical trial to assess the efficacy of 2 weeks of RIC in patients with acute moderate ischemic stroke within 48 h of symptom onset. From December 26, 2018, to April 19, 2021, eligible patients aged 18 years or older with acute moderate ischemic stroke within 48 h of onset at the time of randomization (baseline National Institutes of Health Stroke Scale [NIHSS] scores, 6–16; range, 0–42, with higher scores indicating greater stroke severity) who had been functioning independently in the community before a stroke (modified Rankin Scale [mRS] scores, 0–1; range, 0 [no symptoms] to 6 [death]) were allocated to treatment with either RIC treatment in addition to guideline recommended treatment (such as antiplatelet, anticoagulant, or statins) or only guideline recommended treatment. In the RICAMIS trial, all patients receiving intravenous thrombolysis or other endovascular therapy were excluded. In addition, patients who did not complete the prepositioning intervention were excluded from this study. All patients or their legally authorized representatives signed a written consent form prior to entering the study. The study was registered with ClinicalTrials.gov (NCT03740971). For our analysis, we included patients who had baseline SBP and hypertension history recorded. Baseline blood pressure was automatically measured using an electronic sphygmomanometer in a quiet state by a trained nurse according to a standardized protocol. Hypertension history was defined as a previous diagnosis of hypertension by professional doctors. According to baseline SBP (regardless of antihypertensive medications or not) or hypertension history, eligible patients were divided into SBP <140 mmHg versus SBP ≥140 mmHg group, or hypertension versus nonhypertension group, respectively.

### Procedures

In the secondary analysis, according to hypertension history, patients were divided into two groups: hypertension group and nonhypertension group. To investigate the effect of SBP on efficacy of RIC, patients were divided into two groups: <140 mmHg group and ≥140 mmHg group according to baseline SBP levels. RIC treatment consisted of 5 cycles of cuff inflation (200 mmHg for 5 min) and deflation (for 5 min) for a total procedure time of 50 min, twice daily for 10–14 days. Further details of RIC treatment were contained in previous report.^15^ Neurological status, measured using NIHSS scores, was assessed on admission and after randomization at 7 and 12 days. Follow‐up data, including assessment of prognosis and recurrent events, were collected 90 days after randomization.

### Outcomes

In agreement with RICAMIS trial, the primary outcome was excellent functional outcome at 90 days, defined as an mRS score of 0 to 1. The secondary outcomes included a favorable functional outcome at 90 days, defined as an mRS score of 0 to 2; 90‐day mRS distribution; early neurological deterioration from baseline, defined as an increase of more than 2 points in the NIHSS at Day 7; occurrence of stroke‐associated pneumonia at 12 days; change from baseline in NIHSS at Day 12; time from randomization to occurrence of stroke or other vascular events at 90 days, and occurrence of death within 90 days.

### Statistical analysis

First, patient characteristics summarized after divided by hypertension status. Second, patient characteristics at randomization were summarized after stratification by SBP less than 140 mmHg versus SBP greater than or equal to 140 mmHg. For all comparisons between groups, we respectively calculated the treatment effects of the RIC group compared with the control group. Normality tests (Shapiro–Wilk) were performed on continuous variables. Normally distributed data were expressed as mean ± standard deviation, and non‐normally distributed data were expressed as interquartile range (IQR). Categorical variables were expressed as frequencies and percentages. For normally distributed continuous data, Student's *t*‐test was used to test for significance between the two groups. For non‐normally distributed continuous data, Mann–Whitney U test was used. Categorical variables were compared using chi‐square test or Fisher's exact test. For the treatment effect of outcomes, such as mRS 0–1 and mRS0‐2 at 90 days, occurrence of early neurologic deterioration within 7 days, and occurrence of stroke‐associated pneumonia at 12 days, we estimated the absolute number of events and risk difference with their 95% confidence intervals (CIs). For the treatment effect of mRS score distribution at 90 days, we estimated the odds ratio (OR) with their 95% CIs. For the treatment effect of change in NIHSS score between admission and 12 days, we estimated the geometric mean ratio (GMR) with their 95% CIs. For other secondary outcomes, such as time to occurrence of stroke or other vascular events at 90 days and death at 90 days, we estimated the absolute number of events and hazard ratio (HR) with their 95% CIs.

To test our hypothesis that the association between RIC treatment and functional outcome differed by the first measured blood pressure, we included an interaction term (SBP × treatment), which is the interaction of baseline SBP levels with the treatment group (RIC vs. control). Furthermore, as an exploratory analysis, we also included an interaction term between hypertension × treatment to determine whether a history of hypertension modified the association between RIC treatment and functional outcome. All statistical analyses were performed using the SPSS software (Version 26.0; IBM) and R software (Version 4.2.2; R foundation for Statistical Computing, Vienna, Austria). Two‐sided *p* values below 0.05 were considered statistically significant.

## Results

### Patient characteristics

**Figure 1:**
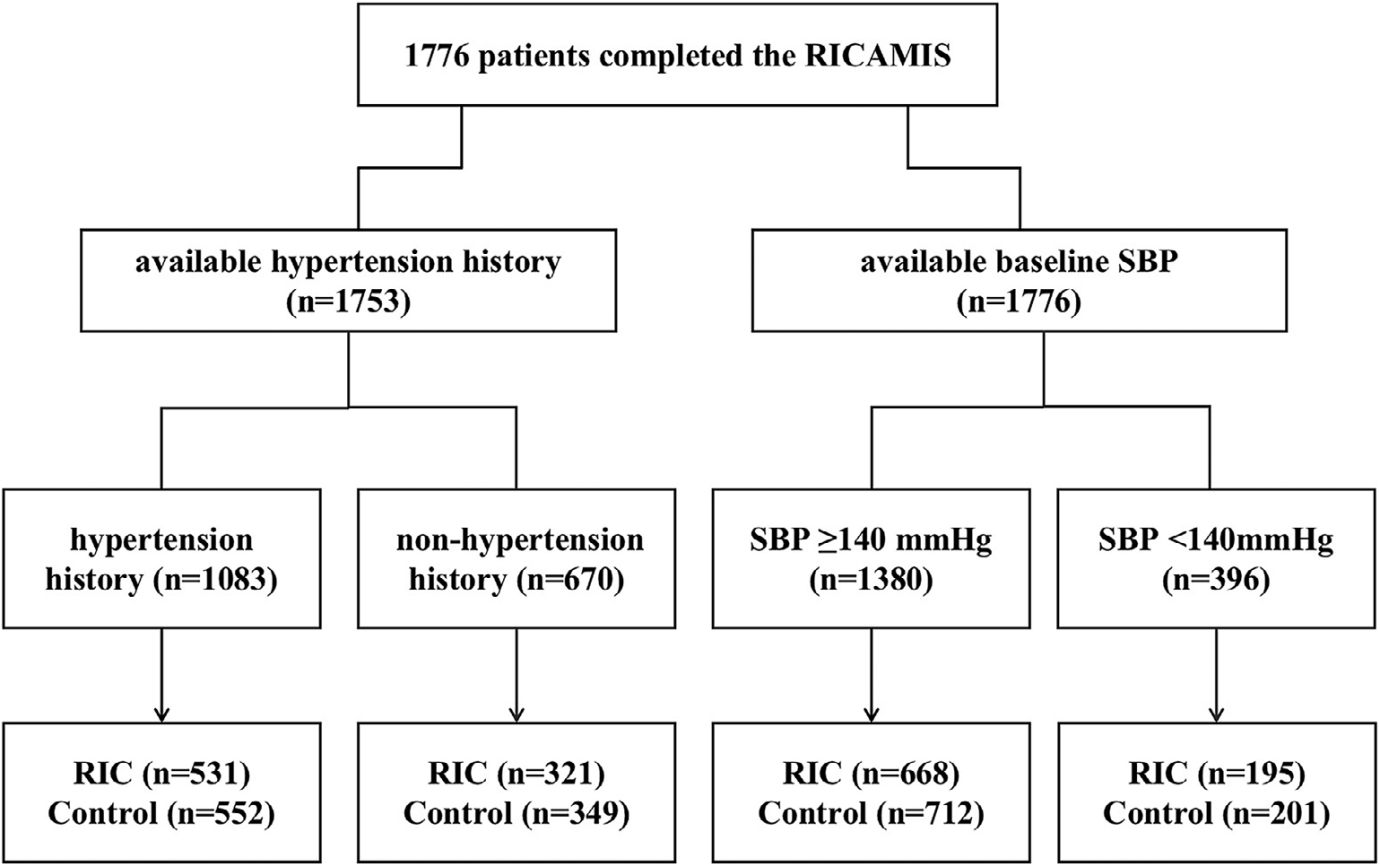
Flowchart of patient inclusion. RIC, remote ischemic conditioning.

A total of 1776 participants from the full analysis set of RICAMIS trial were used for the post hoc analysis, including 863 in the RIC group and 913 in the control group, and 1380 (77.7%) with SBP ≥140 mmHg and 396 (22.3%) with SBP <140 mmHg group (Fig. 1). The median (IQR) age was 65 (58–73) years, and 606 (34.1%) patients were women. In 1753 patients with available hypertension history, 1083 (61.8%) had hypertension history and 670 (38.2%) no hypertension history. The baseline characteristics between the RIC and control groups across the hypertension history or SBP were well balanced (Tables S1 and S2). The baseline characteristics of the hypertension versus nonhypertension group and the SBP <140 mmHg group versus the SBP ≥140 mmHg group were listed in Tables S3 and S4, respectively.

### The effect of hypertension history on the efficacy of RIC

**Figure 2:**
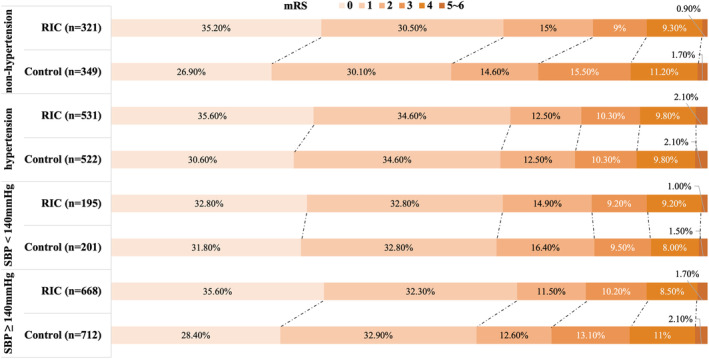
Distribution of modified Rankin Scale (mRS) scores at 90 days. RIC, remote ischemic conditioning.

Table 1 and Figure 2 presented the efficacy of RIC in the hypertension and nonhypertension groups. In the nonhypertension group, the proportion of patients with an mRS score of 0 to 1 at 90 days was significantly higher in the RIC subgroups than that in the control subgroups (65.7% vs. 57.0%; OR 1.45 [95% CI 1.06–1.98], *p* = 0.02). In the hypertension group, proportion of patients with an mRS score of 0 to 1 at 90 days was higher in the RIC subgroup than that in the control subgroup, whereas there was no significant difference (69.1% vs. 65.2%; OR 1.19 [95% CI 0.93–1.54], *p* = 0.17). For the secondary outcomes, the proportion of patients with an mRS of 0 to 2 at 90 days exhibited similar results to the primary outcome (nonhypertension group: 80.7% vs. 71.6%; OR 1.65 [95% CI 1.15–2.38], *p* = 0.006; hypertension group: 79.1% vs. 77.7%; OR 1.09 [95% CI 0.81–1.45]; *p* = 0.58). There were similar results with respect to mRS distribution at 90 days between RIC and control group (nonhypertension group: OR 1.48 [95% CI 0.12–0.66], *p* = 0.005; hypertension group: OR 1.21 [95% CI −0.03–0.40], *p* = 0.09). There were no significant differences according to hypertension history in other secondary outcomes including occurrence of early neurologic deterioration compared with baseline at 7 days, occurrence stroke‐associated pneumonia, change in NIHSS score compared with baseline at 12 days, occurrence of stroke or other vascular events at 90 days, and occurrence of all‐cause death within 90 days. There was no interaction effect of treatment (RIC vs. control) according to different hypertension status (hypertension vs. nonhypertension) for efficacy outcomes.

**Table 1:** Outcomes compared RIC and control efficacy in subgroups according to hypertension history and intervention.

Outcomes	No hypertension history				Hypertension history				*p* for interaction*
	RIC (*N* = 321)	Control (*N* = 349)	Treatment effect	*p* Value	RIC (*N* = 531)	Control (*N* = 522)	Treatment effect	*p* Value	
mRS 0–1 at 90 days	211 (65.7%)	199 (57.0%)	1.45 (1.06 to 1.98)	0.02	367 (69.1%)	360 (65.2%)	1.19 (0.93 to 1.54)	0.17	0.40
mRS 0–2 at 90 days	259 (80.7%)	250 (71.6%)	1.65 (1.15 to 2.38)	0.006	420 (79.1%)	429 (77.7%)	1.09 (0.81 to 1.45)	0.58	0.09
mRS at 90 days			1.48 (1.13 to 1.93)	0.005			1.21 (0.97 to 1.49)	0.09	0.30
0	113 (35.2%)	94 (26.9%)			189 (35.6%)	169 (30.6%)			
1	98 (30.5%)	105 (30.1%)			191 (34.6%)	191 (34.6%)			
2	48 (15.0%)	51 (14.6%)			69 (12.5%)	69 (12.5%)			
3	29 (9.0%)	54 (15.5%)			57 (10.3%)	57 (10.3%)			
4	30 (9.3%)	39 (11.2%)			54 (9.8%)	54 (9.8%)			
5	3 (0.9%)	4 (1.1%)			4 (0.7%)	4 (0.7%)			
6	0	2 (0.6%)			8 (1.4%)	8 (1.4%)			
END within 7 days	28 (7.6)	24 (6.1)	1.29 (0.73 to 2.28)	0.37	49 (9.2)	40 (7.2)	1.30 (0.84 to 2.01)	0.24	0.99
SAP within 12 days	7 (2.2)	9 (2.6)	0.84 (0.31 to 2.29)	0.74	19 (3.6)	10 (1.8)	2.01 (0.93 to 4.37)	0.08	0.14
Change in NIHSS at Day 12 from baseline	−0.3 (−0.6, −0.1)	−0.3 (−0.5, −0.1)	−0.03 (−0.08 to 0.02)	0.28	−0.3 (−0.7, −0.1)	−0.3 (−0.5, −0.1)	0.007 (−0.03 to 0.05)	0.74	0.65
Stroke or other vascular events within 90 days	2 (0.6%)	2 (0.6%)	1.15 (0.16 to 8.30)	0.89	5 (0.9%)	4 (0.7%)	1.42 (0.38 to 5.34)	0.61	0.92
All‐cause death within 90 days	0	2 (0.6%)	NA	0.97	7 (1.3%)	8 (1.4%)	1.10 (0.40 to 3.04)	0.85	0.95

Data were expressed as No. (%) or median (IQR).

END, early neurologic deterioration; IQR, interquartile range; mRS, modified Rankin Scale; NIHSS, National Institute of Health Stroke Scale; RIC, remote ischemic conditioning; SAP, stroke‐associated pneumonia.

* Adjusted for covariates compared between nonhypertension and hypertension subgroups with *p* value <0.05 (Table S3).

### The effect of SBP level at admission on the efficacy of RIC

First, we investigated the effect of SBP as binary categorical variable (SBP ≥140 mmHg vs. SBP <140 mmHg) on efficacy of RIC (Table 2; Fig. 2). In the SBP ≥140 mmHg group, the proportion of patients with 90‐day mRS scores of 0 to 1 was significantly higher in the RIC subgroup than that in control group (68.0% vs. 61.2%; OR 1.34 [95% CI 1.08–1.68], *p* = 0.009). For the secondary outcomes, there was also a significantly higher proportion of patients with an mRS of 0 to 2 at 90 days in the RIC versus control subgroup (SBP ≥140 mmHg: 79.3% vs. 73.9%; OR 1.36 [95% CI 1.06–1.75], *p* = 0.02; SBP <140 mmHg group: 80.5% vs. 80.1%; OR 1.00 [95% CI 0.60–1.66], *p* = 1.00); there were similar results with respect to mRS distribution at 90 days between RIC and control group (SBP ≥140 mmHg: OR 1.36 [95% CI 0.12–0.50], *p* = 0.001; SBP <140 mmHg group: OR 1.04 [95% CI −0.32‐0.40], *p* = 0.82). There were no significant differences in other secondary outcomes, such as occurrence of early neurologic deterioration compared with baseline at 7 days, occurrence stroke‐associated pneumonia, change in NIHSS score compared with baseline at 12 days, occurrence of stroke or other vascular events at 90 days, and occurrence of all‐cause death within 90 days. There was no interaction effect of treatment (RIC vs. control) according to different SBP levels (<140 mmHg vs. ≥140 mmHg) for efficacy outcomes.

**Table 2:** RIC efficacy stratified by SBP 140 mmHg.

Outcomes	SBP <140 mmHg				SBP ≥140 mmHg				*p* for interaction^†^
	RIC (*N* = 195)	Control (*N* = 201)	Treatment effect*	*p* Value	RIC (*N* = 668)	Control (*N* = 712)	Treatment effect	*p* Value	
mRS 0–1 at 90 days	128 (65.6)	130 (64.7)	1.07 (0.70 to 1.62)	0.77	454 (68.0)	436 (61.2)	1.34 (1.08 to 1.68)	0.009	0.31
mRS 0–2 at 90 days	157 (80.5)	163 (80.1)	1.00 (0.60 to 1.66)	1.00	530 (79.3)	526 (73.9)	1.36 (1.06 to 1.75)	0.02	0.24
mRS at 90 days			1.04 (0.73 to 1.49)	0.82			1.36 (1.13 to 1.65)	0.001	0.16
0	64 (32.8)	64 (31.8)			238 (35.6)	202 (28.4)			
1	64 (32.8)	66 (32.8)			216 (32.3)	234 (32.9)			
2	29 (14.9)	33 (16.4)			77 (11.5)	90 (12.6)			
3	18 (9.2)	19 (9.5)			68 (10.2)	93 (13.1)			
4	18 (9.2)	16 (8.0)			57 (8.5)	78 (11.0)			
5	2 (1.0)	1 (0.5)			5 (0.7)	7 (1.0)			
6	0	2 (1.0)			7 (1.0)	8 (1.1)			
END within 7 days	18 (9.2)	11 (5.5)	1.69 (0.77 to 3.70)	0.19	59 (8.8)	53 (7.4)	1.21 (0.82 to 1.78)	0.35	0.44
SAP within 12 days	4 (2.1)	3 (1.5)	1.39 (0.30 to 6.35)	0.67	22 (3.3)	16 (2.2)	1.48 (0.77 to 2.85)	0.24	0.92
Change in NIHSS at Day 12 from baseline	−0.3 (−0.5, −0.1)	−0.3 (−0.6, −0.1)	−0.02 (−0.08 to 0.04)	0.48	−0.3 (−0.7, −0.1)	−0.3 (−0.5, −0.1)	0.03 (−0.009 to 0.06)	0.15	0.16
Stroke or other vascular events within 90 days	0	1 (0.5)	NA	0.62	7 (1.0)	5 (0.7)	1.11 (0.33 to 3.75)	0.87	0.94
All‐cause death within 90 days	0	2 (1.0)	NA	0.48	7 (1.0)	8 (1.1)	0.93 (0.34 to 2.57)	0.89	0.93

Data were expressed as No. (%) or median (IQR).

END, early neurologic deterioration; IQR, interquartile range; mRS, modified Rankin Scale; NIHSS, National Institute of Health Stroke Scale; RIC, remote ischemic conditioning; SAP, stroke‐associated pneumonia.

* Adjusted for current drinker (Table S2).

^†^ Adjusted for current smoker and hypertension (Table S4).

**Figure 3:**
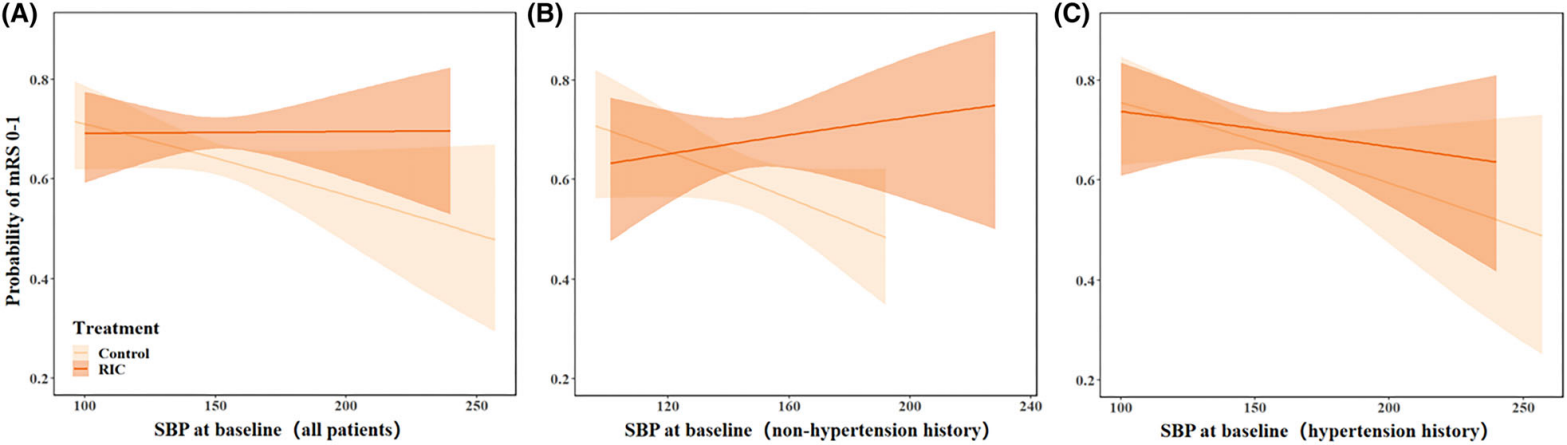
Relationship of baseline systolic blood pressure (SBP) with probability of modified Rankin scale (mRS) scores 0 to 1 at 90 days. The mRS 0–1 with 95% CI for overall patients (A), nonhypertension history (B), and hypertension history (C) over baseline SBP increase. The ranges of the x axes correspond to the lowest and highest SBP value in the data. RIC, remote ischemic conditioning.

Second, we investigated the effect of SBP as continuous variable on efficacy of RIC. We found that as baseline SBP increased, the probability of mRS score of 0 to 1 at 90 days slightly increased in RIC versus control group among all patients (Fig. 3A), which became more obvious in patients without nonhypertension history (Fig. 3B). However, the probability of mRS score of 0 to 1 at 90 days slightly decreased in RIC versus control group in patients with hypertension history as baseline SBP increased (Fig. 3C). Table S5 presented the effect of per 10 mmHg increment in baseline SBP as continuous variable on functional outcome. The results showed that baseline SBP was closely associated with clinical outcome in all patients or control patients (Table S5), but not in RIC patients (Table S5). The similar results were also found in nonhypertension history subgroup (Table S6) and hypertension history subgroup (Table S7).

Third, we investigate the effect of SBP on efficacy of RIC stratified by nonhypertension and hypertension history. In patients with nonhypertension history, the proportion of patients with an mRS score of 0 to 1 or 0–2 at 90 days was significantly higher in the RIC subgroup than that in control group only in SBP ≥140 mmHg group (mRS0–1: 67.8% vs. 55.9%; OR 1.67 [95% CI 1.13–2.46], *p* = 0.01; mRS 0–2 82.5% vs. 68.2%; OR 2.20 [95% CI 1.40–3.46], *p* ≤ 0.001; Table 3), but not in SBP <140 mmHg group (Table 3). Similar results were also observed for mRS distribution at 90 days (OR 1.70 [95% CI 0.18–0.86], *p* = 0.002, Table 3) only in SBP ≥140 mmHg group. However, these findings were not identified in patients with hypertension history (Table S8).

**Table 3:** RIC efficacy in patients without hypertension history stratified by SBP 140 mmHg.

Outcomes	SBP <140 mmHg				SBP ≥140 mmHg				*p* for interaction^‡^
	RIC (*N* = 110)	Control (*N* = 113)	Treatment effect*	*p* Value	RIC (*N* = 211)	Control (*N* = 236)	Treatment effect^†^	*p* Value	
mRS 0–1 at 90 days	68 (61.8)	67 (59.3)	1.15 (0.67 to 1.98)	0.62	143 (67.8)	132 (55.9)	1.67 (1.13 to 2.46)	0.01	0.25
mRS 0–2 at 90 days	85 (77.3)	89 (78.8)	0.98 (0.52 to 1.88)	0.96	174 (82.5)	161 (68.2)	2.20 (1.40 to 3.46)	0.001	0.03
mRS at 90 days			1.15 (0.72 to 1.86)	0.55			1.70 (1.20 to 2.36)	0.002	0.14
0	35 (31.8)	30 (26.5)			78 (37.0)	64 (27.1)			
1	33 (30.0)	37 (32.7)			65 (30.8)	68 (28.8)			
2	17 (15.5)	22 (19.5)			31 (14.7)	29 (12.3)			
3	12 (10.9)	15 (13.3)			17 (8.1)	39 (16.5)			
4	12 (10.9)	7 (6.2)			18 (8.5)	32 (13.6)			
5	1 (0.9)	1 (0.9)			2 (0.9)	3 (1.3)			
6	0	1 (0.9)			0	1 (0.4)			
END within 7 days	10 (9.1)	8 (7.1)	1.29 (0.48 to 3.43)	0.61	18 (8.5)	16 (6.8)	1.26 (0.62 to 2.55)	0.52	0.99
SAP within 12 days	1 (0.9)	2 (1.8)	0.41 (0.04 to 4.91)	0.48	6 (2.8)	7 (3.0)	0.97 (0.32 to 2.94)	0.95	0.63
Change in NIHSS at Day 12 from baseline	−0.3 (−0.6, −0.1)	−0.3 (−0.5, −0.1)	−0.002 (−0.41 to −0.18)	0.95	−0.3 (−0.5, −0.1)	−0.3 (−0.6, −0.1)	−0.05 (−0.11 to 0.01)	0.13	0.39
Stroke or other vascular events within 90 days	0	1 (0.9)	NA	0.62	2 (0.9)	1 (0.4)	2.06 (0.18 to 23.06)	0.56	0.97
All‐cause death within 90 days	0	1 (0.9)	NA	0.62	0	1 (0.4)	NA	0.85	1.00

Data were expressed as No. (%) or median (IQR).

END, early neurologic deterioration; IQR, interquartile range; mRS, modified Rankin Scale; NIHSS, National Institute of Health Stroke Scale; RIC, remote ischemic conditioning; SAP, stroke‐associated pneumonia.

* Adjusted for current drinker (Table S9).

^†^ Adjusted for blood glucose (Table S9).

^‡^ Adjusted for current smoker (Table S10).

Furthermore, we find a significant interaction effect between SBP level (<140 mmHg vs. ≥140 mmHg) and treatment group (RIC vs. control) with an mRS of 0 to 2 at 90 days within nonhypertension patients (*p* for interaction = 0.03), which was not found with respect to other outcomes (Table 3) or in other patients (Tables 1 and 2).

## Discussion

In this post hoc secondary analysis of the RICAMIS trial, we aimed to investigate the effects of hypertension history and baseline SBP on the efficacy of RIC. The results showed that (1) RIC produced a higher probability of excellent functional outcome in the nonhypertension group versus hypertension group and SBP ≥140 mmHg versus <140 mmHg group, but no interaction effect was found between RIC efficacy and hypertension history or baseline SBP; (2) baseline SBP was significantly associated with clinical outcomes in control patients, but the association disappeared in RIC patients; (3) RIC exhibited better benefit in nonhypertension patients with SBP ≥140 mmHg. Collectively, these results suggest that hypertension and baseline SBP do not affect the neuroprotective effect of RIC, but nonhypertension history or high baseline SBP (≥140 mmHg) may be associated with higher probability of excellent functional outcome in patients who received RIC treatment, which was further confirmed in nonhypertension patients with SBP ≥140 mmHg. Furthermore, RIC treatment may attenuate the association of systolic blood pressure with clinical outcomes.

To date, there is no relevant study to investigate the effect of hypertension or SBP on the neuroprotective efficacy of RIC, although their relationship with clinical prognosis has been extensively studied.^25^, ^26^ In this study, we found that RIC showed the potential for better clinical benefit in patients with SBP ≥140 mmHg, although SBP did not affect RIC efficacy. Higher blood pressure during the acute phase after stroke is helpful to prevent further ischemic injury by improving circulation and increasing perfusion, particularly in patients with multiple stenosis of cerebral arteries.^27^, ^28^ Previous clinical study suggested that RIC appeared to improve cerebral perfusion as measured with SPECT imaging, and benefit hemodynamic effects in collaterals.^29^ Thus, better benefit of RIC in patients with baseline SBP ≥140 mmHg may be attributed to a synergistic relationship between higher blood pressure during the acute stages of stroke and the beneficial effects on cerebral perfusion of RIC. In agreement with previous studies,^30^, ^31^ SBP as a continuous variable was found to be associated with clinical outcome in all patients or control patients. Unexpectedly, the association of SBP with clinical outcomes disappeared in RIC patients, suggesting that RIC may attenuate their association, which was never reported previously. As discussed above, this phenomenon may be attributed to the beneficial effect of RIC on cerebral autoregulation, collateral circulation, and cerebral reservoir.^24^, ^30^, ^32^

In this study, we further found that previous hypertension history might be associated with poor efficacy of RIC. We speculate that this may be related to pathological vascular remodeling induced by hypertension.^33^, ^34^ Long‐term hypertension would reduce cerebral flow reserve and cerebral perfusion and impair cerebral autoregulation.^35^, ^36^, ^37^, ^38^ In patients with hypertension history, the improving effect of RIC on cerebral flow reserve and autoregulation may be discounted greatly because of the deterioration of vasoregulation capacity due to long duration hypertension. Furthermore, previous animal experiments suggested that nitric oxide (NO) was a central mediator in the protective effect of RIC.^39^ Hypertension is usually accompanied by endothelial dysfunction which is characterized by decreasing the production or bioavailability of endothelium‐derived relaxing factors, especially NO.^40^, ^41^, ^42^, ^43^ The decreased NO may be another possible explanation of this phenomenon. In addition, given the beneficial effect of normal endothelial function and NO production on cerebral angiogenesis and neuroplasticity,^42^, ^43^ the inhibition of angiogenesis and neuroplasticity due to endothelial dysfunction and decreased NO may further explain the association of hypertension with poor efficacy of RIC, which was also in agreement with the presumed neuroprotective effect of RIC in the RICAMIS trial.

It was worthy to note that in patients with nonhypertension history and baseline SBP ≥140 mmHg, treatment with RIC compared with usual care significantly increased the likelihood of excellent functional outcome at 90 days. This finding was in well agreement with the results of RIC effect in association with SBP and hypertension history. In this population, pathologic vascular remodeling should be slight due to no effect of long‐term hypertension on endothelial injury, and higher SBP may ensure sufficient cerebral perfusion, which may be attributed to better benefit of RIC therapy.

In this study, RIC produced a higher probability of excellent functional outcome in the nonhypertension group versus hypertension group and SBP ≥140 mmHg versus <140 mmHg group, but no interaction effect was found between RIC efficacy and hypertension history or baseline SBP. We contend the lack of an interaction effect between RIC efficacy and hypertension history or baseline SBP may be due to relatively small treatment effect of RIC versus control in overall patients as well as relatively small and imbalanced sample size after stratification. The inference was further supported by the finding in nonhypertension patients with SBP ≥140 mmHg that there was an interaction trend with regard to excellent functional outcome (mRS 0–1) and a significant interaction effect with regard to favorable functional outcome (mRS 0–2).

One main strength of this study was the first report to investigate the association of baseline SBP or hypertension history with RIC efficacy based on a large‐scale, multi‐ center RICAMIS trial. The current finding will be helpful to guide the population selection to investigate RIC efficacy in the future clinical trial design. Our study has several limitations. The main limitation was a secondary post hoc analysis with intrinsic limitation including the relatively small and imbalanced sample size after stratification, which rendered our study underpowered. In addition, the effect of antihypertensive drugs was not excluded due to the lack of these information. Third, RICAMIS study excluded patients with uncontrolled severe hypertension, which may have affected or limited the results of the present study. Fourth, hypertension was defined as SBP ≥140 mmHg or diastolic blood pressure (DBP) ≥90 mmHg according to Chinese guidelines for the management of hypertension,^44^, ^45^ although hypertension was defined as SBP ≥130 mmHg or DBP ≥80 mmHg by 2017 American Heart Association and American College of Cardiology^46^. The impact of the different criteria of hypertension on the current findings should be taken into account. Finally, due to the nature of post hoc analysis, these findings should be explained with caution and warrant confirmation.

## Conclusions

In this post hoc analysis of the RICAMIS trial, hypertension and baseline SBP did not affect the neuroprotective effect of RIC, but they were associated with higher probability of excellent functional outcome in patients with acute moderate ischemic stroke who received RIC treatment.

## Author Contributions

H.S.C contributed to conception and design of the study; Y.C, Y.X.N, Q.W, and Y.N.Z contributed to acquisition and analysis of data; J.R.C and N.N.Z contributed to drafting the text or preparing the figures.

## Conflict of Interest

Nothing to report.

## Supporting information


Table S1.

## Data Availability

The data that support the findings of this study are available from the corresponding author on reasonable request.
