# Supplementary material for: Baseline systolic blood pressure, hypertension history, and efficacy of remote ischemic conditioning
Source: Ann Clin Transl Neurol. 2024 Jun 3;11(7):1703–14. doi: 10.1002/acn3.52077 (PMC11251468; doi:10.1002/acn3.52077)
Supplement: Supplementary file 1 — Table S1. [file ACN3-11-1703-s001.docx]

**Table S1. Baseline Characteristics According to hypertension status and intervention.**

|  | **Non-hypertension history** | | | **Hypertension history** | | |
| --- | --- | --- | --- | --- | --- | --- |
|  | **RIC (N=321)** | **Control (N=349)** | ***P* Value** | **RIC(N=531)** | **Control (N=552)** | ***P* Value** |
| Age, mean (SD), y | 64.7 (10.8) | 65.5 (10.2) | 0.32 | 65.6 (10.3) | 65.2 (10.0) | 0.54 |
| Sex (F), No. (%) | 81 (25.2%) | 97 (27.8%) | 0.45 | 223 (42.0%) | 199 (36.1%) | 0.05 |
| Body mass index, median (IQR), | 23.8(22.0-25.8) | 23.6(22.0-25.5) | 0.64 | 24.4(22.9-26.4) | 24.5(22.9-26.4) | 0.85 |
| Current smoker, No. (%) | 121/311 (38.9%) | 101/335 (30.1%) | 0.12 | 136/518 (26.3%) | 144/532 (27.1%) | 0.77 |
| Current drinker, No. (%) * | 60/314 (19.1%) | 46/341 (13.5%) | 0.18 | 76/524 (14.5%) | 57/534 (10.7%) | 0.06 |
| Comorbidities, No. (%) † | | | | | | |
| Hyperlipidemia | 3/319 (0.9%) | 1/346 (0.3%) | 0.28 | 12/519 (2.3%) | 8/541 (1.5%) | 0.32 |
| Diabetes | 47/321 (14.6%) | 59/347 (83.0%) | 0.40 | 159 (29.9%) | 163/550 (29.6%) | 0.91 |
| Previous ischemic or hemorrhagic stroke | 73/319 (22.9%) | 81/348 (23.3%) | 0.91 | 204/528 (38.4%) | 206/548 (37.6%) | 0.72 |
| Previous transient ischemic attack | 2 (0.6%) | 2 (0.6%) | 0.93 | 9/529 (1.7%) | 8/550 (1.5%) | 0.75 |
| Coronary heart disease | 2/321 (0.6%) | 4/347 (1.2%) | 0.47 | 7/529 (1.3%) | 9/551 (1.6%) | 0.67 |
| Atrial fibrillation | 1 (0.3%) | 2 (0.6%) | 0.61 | 2/530 (0.4%) | 2 (0.4%) | 1.00 |
| OTT, mean (SD), h | 25.2 (12.9) | 24.6 (13.6) | 0.58 | 24.6 (13.4) | 25.3 (13.7) | 0.42 |
| Time to hospital discharge, median (IQR), d | 11 (10,12) | 11 (10,12) | 0.26 | 11 (10,12) | 11 (10,12) | 0.13 |
| Blood pressure at randomization, mean (SD), mmHg | | | | | | |
| Systolic | 146.6 (18.5) | 146.9 (19.3) | 0.79 | 154.2 (18.3) | 154.8 (17.9) | 0.57 |
| ≥140 | 211 (65.7%) | 236 (67.6%) | 0.60 | 449 (84.6%) | 464 (84.1%) | 0.82 |
| Diastolic | 86.8 (11.5) | 87.6 (12.0) | 0.39 | 89.6 (10.9) | 89.8 (10.9) | 0.83 |
| ≥90 | 152 (47.4%) | 176 (50.4%) | 0.43 | 321 (60.5%) | 341 (61.8%) | 0.66 |
| Mean arterial pressure | 106.7 (12.4) | 107.3 (13.0) | 0.51 | 111.1 (11.7) | 111.4 (11.4) | 0.66 |
| ≥102 | 205 (63.9%) | 226 (64.8%) | 0.81 | 421 (79.3%) | 443 (80.3%) | 0.69 |
| Blood glucose, median (IQR), mmol/L | 6.0(5.2-7.4) | 6.1(5.3-7.9) | 0.40 | 6.3(5.5-8.5) | 6.6 (5.5-8.7) | 0.55 |
| NIHSS score at randomization, median (IQR) | 7(6-9) | 7(6-9) | 0.76 | 7(6-9) | 7(6-9) | 0.20 |
| Estimated premorbid function (mRS), No. (%) \|\| | | | | | | |
| No symptoms (score, 0) | 259 (80.7%) | 281 (80.5%) | 0.96 | 380 (71.6%) | 394 (71.4%) | 0.95 |
| Symptoms without any disability (score, 1) | 62 (19.3%) | 68 (19.5%) |  | 151 (28.4%) | 158 (28.6%) |  |
| Presumed stroke cause, No. (%) ¶ | | | | | | |
| Large artery atherosclerosis | 89 (27.7%) | 107 (30.7%) | 0.05 | 137/530 (25.8%) | 178/550 (32.4%) | 0.05 |
| Small artery occlusion | 42 (13.1%) | 66 (18.9%) |  | 80/530 (15.1%) | 92/550 (16.7%) |  |
| Cardioembolic | 4 (1.2%) | 9 (2.6%) |  | 6/530 (1.1%) | 3/550 (0.5%) |  |
| Other determined cause | 6 (1.9%) | 3 (0.9%) |  | 8/530 (1.5%) | 4/550 (0.7%) |  |
| Undetermined cause | 180 (56.1%) | 164 (47.0%) |  | 299/530 (56.4%) | 273/550 (49.6%) |  |

Abbreviations: IQR, interquartile range; SD, Standard Deviation; OTT, time from onset of symptom to remote ischemic conditioning treatment; NIHSS, National Institute of Health Stroke Scale; mRS, modified Rankin Scale.

**Table S2 Baseline Characteristics in patients stratified by SBP 140 mmHg between RIC and control group.**

|  | **SBP＜140 mmHg (N=396)** | | | **SBP≥140 mmHg (N=1380)** | | |
| --- | --- | --- | --- | --- | --- | --- |
|  | **RIC (N=195)** | **Control (N=201)** | ***P* Value** | **RIC(N=668)** | **Control (N=712)** | ***P* Value** |
| Age, mean (SD), y | 66.0 (11.0) | 64.8 (10.3) | 0.24 | 65.1 (10.3) | 65.5 (10.0) | 0.46 |
| Sex (F), No. (%) | 58 (29.7) | 64 (31.8) | 0.67 | 249 (37.3) | 235 (33.0) | 0.10 |
| Body mass index, median (IQR), | 23.9 (21.9-26.0) | 23.8 (22.3-25.4) | 0.96 | 24.5 (4.1) | 24.4 (3.0) | 0.86 |
| Current smoker, No. (%) | 76/189 (40.2) | 61/191 (31.9) | 0.09 | 183/650 (28.2) | 185/687 (26.9) | 0.62 |
| Current drinker, No. (%) * | 36.191 (18.8) | 20/197 (10.2) | 0.02 | 101/657 (15.4) | 83/690 (12.0) | 0.07 |
| Comorbidities, No. (%) † | | | | | | |
| Hypertension | 82/192 (42.7) | 88 (43.8) | 0.83 | 449/660 (68.0) | 464/700 (66.3) | 0.49 |
| Dyslipidemia | 6/189 (3.2) | 3/197 (1.5) | 0.46 | 9/657 (1.4) | 6/701 (0.9) | 0.37 |
| Diabetes | 40/194 (20.6) | 48 (23.9) | 0.47 | 168 (25.1) | 175/707 (24.8) | 0.87 |
| Previous ischemic or hemorrhagic stroke | 73/195 (37.4) | 60/199 (30.2) | 0.13 | 207/663 (31.2) | 229/708 (32.3) | 0.66 |
| Previous transient ischemic attack | 1 (0.5) | 2 (1.0) | 1.00 | 10/666 (1.5) | 9/710 (1.3) | 0.71 |
| Coronary heart disease | 4 (2.1) | 3/199 (1.5) | 0.98 | 5/666 (0.8) | 10/711 (1.4) | 0.24 |
| Atrial fibrillation | 0 | 2 (1.0) | 0.49 | 4/667 (0.6) | 2 (0.3) | 0.62 |
| OTT, median (IQR), h | 26.2 (16.5-34.5) | 24.3 (13.4-33.2) | 0.10 | 25.1 (12.3-34.3) | 25.3 (12.3-36.1) | 0.28 |
| Time to hospital discharge, median (IQR), d | 10 (9-12) | 11 (10-12) | 0.12 | 11 (10-12) | 11 (10-12) | 0.26 |
| Blood pressure at randomization, mean (SD), mmHg | | | | | | |
| Systolic | 127.3 (8.1) | 126.9 (9.4) | 0.62 | 158.3 (14.7) | 158.8 (14.2) | 0.50 |
| Diastolic | 80.1 (9.7) | 80.2 (10.4) | 0.88 | 91.0 (10.4) | 91.4 (10.4) | 0.50 |
| Mean arterial pressure | 95.8 (7.9) | 95.8 (8.8) | 0.96 | 113.5 (10.1) | 113.9 (9.8) | 0.43 |
| Blood glucose, median (IQR), mmol/L | 6.2 (5.4-7.8) | 6.1 (5.2-8.0) | 0.56 | 6.2 (5.4-8.2) | 6.4 (5.5-8.5) | 0.17 |
| NIHSS score at randomization, median (IQR) | 7 (6-10) | 7 (6-9) | 0.59 | 7 (6-9) | 7 (6-9) | 0.51 |
| Estimated premorbid function (mRS), No. (%) \|\| | | | | | | |
| No symptoms (score, 0) | 154 (76.6) | 144 (73.8) | 0.52 | 503 (75.3) | 531 (74.6) | 0.76 |
| Symptoms without any disability (score, 1) | 51 (26.2) | 47 (23.4) |  | 165 (24.7) | 181 (25.4) |  |
| Presumed stroke cause, No. (%) ¶ | | | | | | |
| Large artery atherosclerosis | 56 (28.7) | 58 (28.9) | 0.05 | 173 (25.9) | 229 (32.3) | 0.05 |
| Small artery occlusion | 3 (1.5) | 5 (2.5) |  | 7 (1.0) | 7 (1.0) |  |
| Cardioembolic | 22 (11.3) | 43 (21.4) |  | 101 (15.1) | 118 (16.6) |  |
| Other determined cause | 3 (1.5) | 1 (0.5) |  | 11 (1.6) | 7 (1.0) |  |
| Undetermined cause | 111 (56.9) | 94 (46.8) |  | 375 (56.2) | 349 (29.2) |  |

Abbreviations: IQR, interquartile range; SD, Standard Deviation; OTT, time from onset of symptom to remote ischemic conditioning treatment; NIHSS, National Institute of Health Stroke Scale; mRS, modified Rankin Scale.

**Table S3. Baseline Characteristics According to hypertension history.**

|  | **No hypertension (n=670)** | **Hypertension (n=1083)** | ***P* Value** |
| --- | --- | --- | --- |
| Age, mean (SD), y | 65.1 (10.5) | 65.4 (10.1) | 0.54 |
| Sex (F), No. (%) | 178 (26.6) | 422 (39.0) | ＜0.001 |
| Body mass index, median (IQR) | 23.8 (22.0,25.8) | 24.3 (22.9,26.2) | 0.85 |
| Current smoker, No. (%) | 222/646 (34.4) | 280/1050 (26.7) | 0.001 |
| Current drinker, No. (%) * | 106/655 (16.2) | 133/1058 (12.6) | 0.04 |
| Comorbidities, No. (%) † | | | |
| Hyperlipidemia | 4/665 (0.6) | 20/1060 (1.9) | 0.03 |
| Diabetes | 106/668 (15.9) | 322/1081 (29.8) | ＜0.001 |
| Previous ischemic or hemorrhagic stroke | 154/667 (23.1) | 410/1076 (38.1) | ＜0.001 |
| Previous transient ischemic attack | 4 (0.6) | 17/1079 (1.6) | 0.07 |
| Coronary heart disease | 6/668 (0.9) | 16 (1.5) | 0.29 |
| Atrial fibrillation | 3 (0.4) | 4/1082 (0.4) | 1.00 |
| OTT, mean (SD), h | 25.5 (13.2-34.9) | 25.1(13.0-35.1) | 0.97 |
| Time to hospital discharge, median (IQR), d | 11 (10-12) | 11 (10-12) | 0.32 |
| Blood pressure at randomization, mean (SD), mmHg | | | |
| Systolic | 146.8 (18.9) | 154.5 (18.1) | 0.57 |
| Diastolic | 87.2 (11.8) | 89.7 (10.9) | 0.83 |
| Mean arterial pressure | 107.0 (12.7) | 111.3 (11.5) | 0.66 |
| Blood glucose, median (IQR), mmol/L | 6.1 (5.3-7.7) | 6.5 (5.5-8.6) | 0.55 |
| NIHSS score at randomization, median (IQR) | 7 (6-9) | 7 (6-9) | 0.20 |
| Estimated premorbid function (mRS), No. (%) \|\| | | | |
| No symptoms (score, 0) | 540 (80.6) | 774 (71.5) | ＜0.001 |
| Symptoms without any disability (score, 1) | 130 (19.4) | 309 (28.5) |  |
| Presumed stroke cause, No. (%) ¶ | | | |
| Large artery atherosclerosis | 196 (29.3) | 315/1080 (29.2) | 0.35 |
| Small artery occlusion | 13 (1.9) | 9/1080 (0.8) |  |
| Cardioembolic | 108 (16.1) | 172/1080 (15.9) |  |
| Other determined cause | 9 (1.3) | 12/1080 (1.1) |  |
| Undetermined cause | 344 (51.3) | 572/1080 (53.0) |  |

Abbreviations: IQR, interquartile range; SD, Standard Deviation; OTT, time from onset of symptom to remote ischemic conditioning treatment; NIHSS, National Institute of Health Stroke Scale; mRS, modified Rankin Scale.

**Table S4 Baseline Characteristics in patients stratified by SBP 140 mmHg.**

|  | **SBP＜140 mmHg (N=396)** | **SBP≥140 mmHg (N=1380)** | ***P* Value** |
| --- | --- | --- | --- |
| Age, mean (SD), y | 65.4 (10.7) | 65.3 (10.1) | 0.91 |
| Sex (F), No. (%) | 122 (30.8) | 484 (35.1) | 0.12 |
| Body mass index, median (IQR) | 23.9 (22.0-25.8) | 24.2 (22.7-26.1) | 0.82 |
| Current smoker, No. (%) | 137/380 (36.1) | 368/1337 (27.5) | 0.001 |
| Current drinker, No. (%) * | 56/388 (14.4) | 184/1347 (13.7) | 0.70 |
| Comorbidities, No. (%) † | | | |
| Hypertension | 170/393 (43.3) | 913/1360 (67.1) | ＜0.001 |
| Hyperlipidemia | 9/386 (2.3) | 15/1358 (1.1) | 0.07 |
| Diabetes | 88/395 (22.3) | 343/1375 (24.9) | 0.29 |
| Previous ischemic or hemorrhagic stroke | 133/394 (33.8) | 436/1371 (31.8) | 0.46 |
| Previous transient ischemic attack | 3 (0.8) | 19/1376 (1.4) | 0.32 |
| Coronary heart disease | 7/394 (1.8) | 15/1377 (1.1) | 0.28 |
| Atrial fibrillation | 2 (0.5) | 6/1379 (0.4) | 0.86 |
| OTT, mean (SD), h | 25.0 (13.0) | 24.9 (13.6) | 0.86 |
| Time to hospital discharge, median (IQR), d | 11 (9-12) | 11 (10-12) | 0.05 |
| Blood pressure at randomization, mean (SD), mmHg | | | |
| Systolic | 127.1 (8.8) | 158.6 (14.5) | ＜0.001 |
| Diastolic | 80.2 (10.0) | 91.2 (10.4) | ＜0.001 |
| Mean arterial pressure | 95.8 (8.4) | 113.7 (10.0) | ＜0.001 |
| Blood glucose, median (IQR), mmol/L | 6.1 (5.3-8.0) | 6.3 (5.4-8.4) | 0.35 |
| NIHSS score at randomization, median (IQR) | 7 (6-9) | 7 (6-9) | 0.40 |
| Estimated premorbid function (mRS), No. (%) \|\| | | | |
| No symptoms (score, 0) | 298 (75.3) | 1034 (74.9) | 0.81 |
| Symptoms without any disability (score, 1) | 98 (24.7) | 346 (25.1) |  |
| Presumed stroke cause, No. (%) ¶ | | | |
| Large artery atherosclerosis | 114 (28.8) | 402/1377 (29.2) | 0.59 |
| Small artery occlusion | 8 (2.0) | 14/1377 (1.0) |  |
| Cardioembolic | 65 (16.4) | 219/1377 (15.9) |  |
| Other determined cause | 4 (1.0) | 18/1377 (1.3) |  |
| Undetermined cause | 205 (51.8) | 724/1377 (52.6) |  |

Abbreviations: IQR, interquartile range; SD, Standard Deviation; OTT, time from onset of symptom to remote ischemic conditioning treatment; NIHSS, National Institute of Health Stroke Scale; mRS, modified Rankin Scale.

**Table S5.** **The effect of per 10mmHg increment in baseline SBP as continuous variable on functional outcome.**

| **Outcomes** | **Overall patients (n=1776)** | | **RIC patients (n=863)** | | **Control patients (n=913)** | |
| --- | --- | --- | --- | --- | --- | --- |
|  | **Adjusted OR, 95% (CI)** | **P Value** | **Adjusted OR, 95% (CI)** | **P Value** | **Adjusted OR, 95% (CI)** | **P Value** |
| mRS 0-1 at 90 d | 0.95 (0.90 to 1.01) | 0.09 | 1.00 (0.92 to 1.08) | 0.96 | 0.92 (0.85 to 0.99) | 0.03 |
| mRS 0-2 at 90 d | 0.92 (0.86 to 0.98) | 0.009 | 1.00 (0.91 to 1.10) | 0.98 | 0.85 (0.78 to 0.93) | 0.001 |
| mRS at 90 d | 1.05 (1.01 to 1.10) | 0.03 | 1.00 (0.94 to 1.07) | 0.92 | 1.10 (1.03 to 1.18) | 0.003 |
| END within 7 d | 1.08 (0.99 to 1.18) | 0.10 | 0.98 (0.86 to 1.11) | 0.74 | 1.22 (1.06 to 1.40) | 0.005 |
| SAP within 12 d | 1.15 (0.98 to 1.35) | 0.09 | 1.14 (0.92 to 1.41) | 0.22 | 1.17 (0.91 to 1.50) | 0.22 |
| Change in NIHSS at Day 12 from baseline | 0.01 (0.002 to 0.02) | 0.01 | 0.004 (-0.008 to 0.02) | 0.49 | 0.02 (0.005 to 0.03) | 0.003 |
| Stroke or other vascular events within 90 d | 1.20 (0.91 to 1.57) | 0.21 | 1.24 (0.84 to 1.81) | 0.28 | 1.15 (0.76 to 1.74) | 0.51 |
| All-cause death within 90 d | 1.26 (0.99 to 1.61) | 0.06 | 1.27 (0.85 to 1.91) | 0.25 | 1.24 (0.89 to 1.73) | 0.21 |

Note: Data were expressed as No. (%) or median (IQR). Abbreviations: IQR, interquartile range; mRS, modified Rankin Scale; NIHSS, National Institute of Health Stroke Scale; RIC, remote ischemic conditioning; SAP, stroke associated pneumonia; END, early neurologic deterioration. Adjusted for key prognostic covariates (age, sex, premorbid function [mRS score, 0 or 1], NIHSS score at randomization, time from onset of symptom to randomization) and hypertension.

**Table S6. The effect of per 10mmHg increment in baseline SBP as continuous variable on functional outcome within non-hypertension patients.**

| **Outcomes** | **Overall patients (n=670)** | | **RIC patients (n=321)** | | **Control patients (n=349)** | |
| --- | --- | --- | --- | --- | --- | --- |
|  | **Adjusted OR, 95% (CI)** | **P Value** | **Adjusted OR, 95% (CI)** | **P Value** | **Adjusted OR, 95% (CI)** | **P Value** |
| mRS 0-1 at 90 d | 0.94 (0.86 to 1.02) | 0.14 | 1.00 (0.87 to 1.14) | 0.97 | 0.89 (0.79 to 1.01) | 0.07 |
| mRS 0-2 at 90 d | 0.91 (0.83 to 1.01) | 0.08 | 1.12 (0.94 to 1.32) | 0.21 | 0.80 (0.70 to 0.92) | 0.002 |
| mRS at 90 d | 1.05 (0.99 to 1.15) | 0.09 | 1.01 (0.90 to 1.13) | 0.90 | 1.12 (1.01 to 1.23) | 0.03 |
| END within 7 d | 1.07 (0.92 to 1.24) | 0.39 | 0.97 (0.78 to 1.21) | 0.79 | 1.20 (0.95 to 1.50) | 0.12 |
| SAP within 12 d | 1.21 (0.94 to 1.57) | 0.14 | 1.25 (0.88 to 1.78) | 0.21 | 1.12 (0.79 to 1.60) | 0.52 |
| Change in NIHSS at Day 12 from baseline | 0.01 (-0.003 to 0.02) | 0.12 | -0.002 (-0.02 to 0.02) | 0.82 | 0.02 (0.004 to 0.04) | 0.02 |
| Stroke or other vascular events within 90 d | 0.97 (0.57 to 1.66) | 0.92 | 1.23 (0.64 to 2.36) | 0.54 | 0.76 (0.36 to 1.60) | 0.47 |
| All-cause death within 90 d | 0.90 (0.44 to 1.85) | 0.78 | NA | NA | 0.87 (0.46 to 1.67) | 0.68 |

Note: Data were expressed as No. (%) or median (IQR). Abbreviations: IQR, interquartile range; mRS, modified Rankin Scale; NIHSS, National Institute of Health Stroke Scale; RIC, remote ischemic conditioning; SAP, stroke associated pneumonia; END, early neurologic deterioration. Adjusted for key prognostic covariates (age, sex, premorbid function [mRS score, 0 or 1], NIHSS score at randomization, time from onset of symptom to randomization).

**Table S7. The effect of per 10mmHg increment in baseline SBP as continuous variable on functional outcome within previously diagnosed hypertension patients.**

| **Outcomes** | **Overall patients (n=1083)** | | **RIC patients (n=531)** | | **Control patients (n=552)** | |
| --- | --- | --- | --- | --- | --- | --- |
|  | **Adjusted OR, 95% (CI)** | **P Value** | **Adjusted OR, 95% (CI)** | **P Value** | **Adjusted OR, 95% (CI)** | **P Value** |
| mRS 0-1 at 90 d | 0.97 (0.90 to 1.05) | 0.43 | 1.00 (0.90 to 1.12) | 0.97 | 0.94 (0.84 to 1.05) | 0.26 |
| mRS 0-2 at 90 d | 0.92 (0.85 to 1.00) | 0.06 | 0.95 (0.85 to 1.08) | 0.45 | 0.90 (0.80 to 1.01) | 0.07 |
| mRS at 90 d | 1.04 (0.98 to 1.11) | 0.18 | 0.99 (0.91 to 1.08) | 0.81 | 1.09 (1.00 to 1.19) | 0.04 |
| END within 7 d | 1.09 (0.97 to 1.22) | 0.16 | 0.99 (0.84 to 1.16) | 0.85 | 1.24 (1.04 to 1.47) | 0.02 |
| SAP within 12 d | 1.10 (0.89 to 1.36) | 0.37 | 1.07 (0.83 to 1.40) | 0.60 | 1.18 (0.82 to 1.68) | 0.37 |
| Change in NIHSS at Day 12 from baseline | 0.01 (0.000 to 0.02) | 0.05 | 0.008 (-0.008 to 0.02) | 0.32 | 0.02 (0.000 to 0.03) | 0.04 |
| Stroke or other vascular events within 90 d | 1.30 (0.95 to 1.79) | 0.10 | 1.29 (0.79 to 2.11) | 0.32 | 1.35 (0.83 to 2.21) | 0.23 |
| All-cause death within 90 d | 1.32 (1.02 to 1.71) | 0.03 | 1.27 (0.85 to 1.90) | 0.25 | 1.39 (0.95 to 2.04) | 0.09 |

Note: Data were expressed as No. (%) or median (IQR). Abbreviations: IQR, interquartile range; mRS, modified Rankin Scale; NIHSS, National Institute of Health Stroke Scale; RIC, remote ischemic conditioning; SAP, stroke associated pneumonia; END, early neurologic deterioration. Adjusted for key prognostic covariates (age, sex, premorbid function [mRS score, 0 or 1], NIHSS score at randomization, time from onset of symptom to randomization).

**Table S8. Outcomes compared RIC and control efficacy within previously diagnosed hypertension patients stratified by SBP 140 mmHg.**

| **Outcomes** | **SBP＜140 mmHg** | | | | **SBP≥140 mmHg** | | | | ***P* for Interaction†** |
| --- | --- | --- | --- | --- | --- | --- | --- | --- | --- |
|  | **RIC (N=82)** | **Control (N=88)** | **Treatment effect** | ***P* Value** | **RIC (N=449)** | **Control (N=464)** | **Treatment effect*** | ***P* Value** |  |
| mRS 0-1 at 90 d | 59 (72.0%) | 63 (71.6%) | 1.02 (0.52 to 1.99) | 0.96 | 308 (68.6%) | 297 (64%) | 1.27 (0.96 to 1.67) | 0.09 | 0.61 |
| mRS 0-2 at 90 d | 70 (85.4%) | 74 (84.1%) | 1.10 (0.48 to 2.55) | 0.82 | 350 (78.0%) | 355 (76.5%) | 1.13 (0.82 to 1.54) | 0.46 | 0.96 |
| mRS at 90 d |  |  | 0.95 (0.55 to 1.65) | 0.86 |  |  | 1.26 (0.99 to 1.60) | 0.06 | 0.36 |
| 0 | 29 (35.4%) | 34 (38.6%) |  |  | 160 (35.6%) | 135 (29.1%) |  |  |  |
| 1 | 30 (36.6%) | 29 (33.0%) |  |  | 148 (33.0%) | 162 (34.9%) |  |  |  |
| 2 | 11 (13.4%) | 11 (12.5%) |  |  | 43 (9.6%) | 58 (12.5%) |  |  |  |
| 3 | 5 (6.1%) | 4 (4.5%) |  |  | 50 (11.1%) | 53 (11.4%) |  |  |  |
| 4 | 6 (7.3%) | 9 (10.2%) |  |  | 38 (8.5%) | 45 (9.7%) |  |  |  |
| 5 | 1 (1.2%) | 0 |  |  | 3 (0.7%) | 4 (0.9%) |  |  |  |
| 6 | 0 | 1 (1.1%) |  |  | 7 (1.6%) | 7 (1.5%) |  |  |  |
| END within 7 d | 8 (9.8%) | 3 (3.4%) | 3.06 (0.78 to 11.97) | 0.11 | 41 (9.1%) | 37 (8.0%) | 1.13 (0.71 to 1.80) | 0.61 | 0.18 |
| SAP within 12 d | 3 (3.7%) | 1 (1.1%) | 3.30 (0.34 to 32.42) | 0.31 | 16 (3.6%) | 9 (1.9%) | 1.98 (0.86 to 4.55) | 0.11 | 0.71 |
| Change in NIHSS at Day 12 from baseline | -0.3 (-0.8,-0.1) | -0.3 (-0.8,-0.1) | -0.05 (-0.15 to 0.04) | 0.28 | -0.3 (-0.7,-0.1) | -0.3 (-0.5,-0.1) | 0.02 (-0.02 to 0.07) | 0.32 | 0.20 |
| Stroke or other vascular events within 90 d | 0 | 0 | NA | NA | 5 (1.1%) | 4 (0.9%) | 1.28 (0.34 to 4.78) | 0.72 | 1.00 |
| All-cause death within 90 d | 0 | 1 (1.1) | NA | 0.62 | 7 (1.6%) | 7 (1.5%) | 1.07 (0.37 to 3.06) | 0.90 | 0.96 |

Note: Data were expressed as No. (%) or median (IQR). Abbreviations: IQR, interquartile range; mRS, modified Rankin Scale; NIHSS, National Institute of Health Stroke Scale; RIC, remote ischemic conditioning; SAP, stroke associated pneumonia; END, early neurologic deterioration. *****Adjusted for sex (Table S11). **†**Adjusted for body mass index (Table S12

**Table S9. Baseline Characteristics of no hypertension patients stratified by SBP 140 mmHg.**

|  | **SBP＜140 mmHg** | | | **SBP≥140 mmHg** | | |
| --- | --- | --- | --- | --- | --- | --- |
|  | **RIC (N=110)** | **Control (N=113)** | ***P* Value** | **RIC (N=211)** | **Control (N=236)** | ***P* Value** |
| Age, mean (SD), y | 65.7 (10.9) | 64.9 (10.8) | 0.58 | 64.2 (10.7) | 65.8 (9.9) | 0.10 |
| Sex (F), No. (%) | 30 (27.3) | 31 (27.4) | 0.98 | 51 (24.2) | 66 (28.0) | 0.36 |
| Body mass index, median (IQR), | 24.0 (21.5-26.0) | 23.1 (22.0-25.2) | 0.54 | 23.7 (22.1-25.7) | 23.9 (22.0-25.6) | 0.95 |
| Current smoker, No. (%) | 49/106 (46.2) | 37/108 (34.3) | 0.07 | 72/205 (35.1) | 64/227 (28.2) | 0.12 |
| Current drinker, No. (%) * | 21/107 (19.6) | 10/112 (8.9) | 0.02 | 39/207 (18.8) | 36/229 (15.7) | 0.39 |
| Comorbidities, No. (%) † | | | | | | |
| Hyperlipidemia | 2/108 (1.9) | 1/111 (0.9) | 0.98 | 1 (0.5) | 0 | 0.96 |
| Diabetes | 18 (16.4) | 17 (15.0) | 0.79 | 29 (13.7) | 42 (17.9) | 0.23 |
| Previous ischemic or hemorrhagic stroke | 30 (27.3) | 26/112 (23.2) | 0.49 | 43/209 (20.6) | 55 (23.3) | 0.49 |
| Previous transient ischemic attack | 0 | 2 (1.8) | 0.49 | 2 (0.9) | 0 | 0.43 |
| Coronary heart disease | 1 (0.9) | 3/112 (2.7) | 0.63 | 1 (0.5) | 1 (0.4) | 1.00 |
| Atrial fibrillation | 0 | 2 (1.8) | 0.49 | 1 (0.5) | 0 | 0.47 |
| OTT, median (IQR), h | 26.9 (20.1-34.7) | 24.5 (9.8-34.1) | 0.05 | 24.1 (13.2) | 25.1 (13.7) | 0.43 |
| Time to hospital discharge, mean (SD), d | 10.2 (2.7) | 10.8 (1.8) | 0.51 | 10.5 (2.6) | 10.9 (1.5) | 0.07 |
| Blood pressure at randomization, median (IQR), mmHg | | | | | | |
| Systolic | 130 (125-132) | 130 (120-132) | 0.09 | 156.3 (14.6) | 157.3 (13.1) | 0.43 |
| Diastolic | 80 (72-85) | 80 (72-85) | 0.83 | 90.6 (10.4) | 91.6 (10.8) | 0.29 |
| Mean arterial pressure | 96.7 (91.9-100.8) | 95.0 (90.3-100.0) | 0.36 | 112.5 (10.2) | 113.5 (9.9) | 0.27 |
| Blood glucose, median (IQR), mmol/L | 6.2 (5.3-8.1) | 5.8 (5.0-6.9) | 0.06 | 6.0 (5.2-7.2) | 6.3 (5.4-8.2) | 0.02 |
| NIHSS score at randomization, median (IQR) | 7 (6-10) | 7 (6-9) | 0.31 | 7 (6-9) | 7 (6-9) | 0.26 |
| Estimated premorbid function (mRS), No. (%) \|\| | | | | | | |
| No symptoms (score, 0) | 87 (79.1) | 90 (79.6) | 0.92 | 172 (81.5) | 191 (80.9) | 0.88 |
| Symptoms without any disability (score, 1) | 23 (20.9) | 23 (20.4) |  | 39 (18.5) | 45 (19.1) |  |
| Presumed stroke cause, No. (%) ¶ | | | | | | |
| Large artery atherosclerosis | 34 (30.9) | 36 (31.9) | 0.21 | 55 (26.1) | 71 (30.1) | 0.25 |
| Small artery occlusion | 2 (1.8) | 4 (3.5) |  | 2 (0.9) | 5 (2.1) |  |
| Cardioembolic | 14 (12.7) | 26 (23.0) |  | 28 (13.3) | 40 (16.9) |  |
| Other determined cause | 1 (0.9) | 1 (0.9) |  | 5 (2.4) | 2 (0.8) |  |
| Undetermined cause | 59 (53.6) | 46 (40.7) |  | 121 (57.3) | 118 (50.0) |  |

Abbreviations: IQR, interquartile range; SD, Standard Deviation; OTT, time from onset of symptom to remote ischemic conditioning treatment; NIHSS, National Institute of Health Stroke Scale; mRS, modified Rankin Scale.

**Table S10. Baseline Characteristics of without hypertension history patients stratified by SBP 140 mmHg.**

|  | **SBP＜140 mmHg (N=223)** | **SBP≥140 mmHg (N=447)** | ***P* Value** |
| --- | --- | --- | --- |
| Age, mean (SD), y | 65.3 (10.9) | 65.0 (10.3) | 0.82 |
| Sex (F), No. (%) | 61 (27.4) | 117 (26.2) | 0.75 |
| Body mass index, median (IQR) | 23.6 (22.0-25.4) | 23.9 (22.0-25.8) | 0.26 |
| Current smoker, No. (%) | 86/214 (40.2) | 136/432 (31.5) | 0.03 |
| Current drinker, No. (%) * | 31/219 (14.2) | 75/436 (17.2) | 0.32 |
| Comorbidities, No. (%) † | | | |
| Hyperlipidemia | 3/219 (1.4) | 1/446 (0.2) | 0.21 |
| Diabetes | 35/223 (15.7) | 71/445 (16.0) | 0.93 |
| Previous ischemic or hemorrhagic stroke | 56/222 (25.2) | 98/445 (22.0) | 0.36 |
| Previous transient ischemic attack | 2 (0.9) | 2 (0.4) | 0.48 |
| Coronary heart disease | 4/222 (1.8) | 2/446 (0.4) | 0.19 |
| Atrial fibrillation | 2 (0.9) | 1 (0.2) | 0.54 |
| OTT, mean (SD), h | 25.4 (13.0) | 24.6 (13.5) | 0.46 |
| Time to hospital discharge, median (IQR), d | 10 (10-12) | 11 (10-12) | 0.34 |
| Blood pressure at randomization, mean (SD), mmHg | | | |
| Systolic | 126.6 (9.0) | 156.8 (13.8) | ＜0.001 |
| Diastolic | 79.3 (9.8) | 91.1 (10.6) | ＜0.001 |
| Mean arterial pressure | 95.0 (8.4) | 113.0 (10.0) | ＜0.001 |
| Blood glucose, median (IQR), mmol/L | 6.3 (5.4-8.2) | 6.5 (5.5-8.8) | 0.30 |
| NIHSS score at randomization, median (IQR) | 7 (6-9) | 7 (6-10) | 0.63 |
| Estimated premorbid function (mRS), No. (%) \|\| | | | |
| No symptoms (score, 0) | 177 (79.4) | 363 (81.2) | 0.57 |
| Symptoms without any disability (score, 1) | 46 (20.6) | 84 (18.8) |  |
| Presumed stroke cause, No. (%) ¶ | | | |
| Large artery atherosclerosis | 70 (31.4) | 126 (28.2) | 0.43 |
| Small artery occlusion | 6 (2.7) | 7 (1.6) |  |
| Cardioembolic | 40 (17.9) | 68 (15.2) |  |
| Other determined cause | 2 (0.9) | 7 (1.6) |  |
| Undetermined cause | 105 (47.1) | 239 (53.5) |  |

Abbreviations: IQR, interquartile range; SD, Standard Deviation; OTT, time from onset of symptom to remote ischemic conditioning treatment; NIHSS, National Institute of Health Stroke Scale; mRS, modified Rankin Scale.

**Table S11. Baseline Characteristics of previously diagnosed hypertension patients stratified by SBP 140 mmHg.**

|  | **SBP＜140 mmHg** | | | **SBP≥140 mmHg** | | |
| --- | --- | --- | --- | --- | --- | --- |
|  | **RIC (N=82)** | **Control (N=88)** | ***P* Value** | **RIC (N=449)** | **Control (N=464)** | ***P* Value** |
| Age, mean (SD), y | 66.5 (11.4) | 64.6 (9.7) | 0.25 | 65.5 (10.1) | 65.4 (10.1) | 0.87 |
| Sex (F), No. (%) | 27 (32.9) | 33 (37.5) | 0.53 | 196 (43.7) | 166 (35.8) | 0.02 |
| Body mass index, median (IQR), | 24.0 (22.1-26.1) | 24.1 (22.9-25.7) | 0.60 | 24.5 (22.9-26.4) | 24.5 (22.9-26.7) | 0.93 |
| Current smoker, No. (%) | 26 (32.5) | 24 (28.9) | 0.62 | 110 (25.1) | 120 (26.7) | 0.58 |
| Current drinker, No. (%) * | 14 (17.3) | 10 (11.8) | 0.31 | 62 (14.0) | 47 (10.5) | 0.11 |
| Comorbidities, No. (%) † | | | | | | |
| Hyperlipidemia | 4 (5.1) | 2 (2.3) | 0.60 | 8 (1.8) | 6 (1.3) | 0.55 |
| Diabetes | 20 (24.4) | 31 (35.2) | 0.12 | 139 (31.0) | 132 (28.6) | 0.43 |
| Previous ischemic or hemorrhagic stroke | 42 (51.2) | 34 (39.1) | 0.11 | 162 (36.3) | 172 (37.3) | 0.76 |
| Previous transient ischemic attack | 1 (1.2) | 0 | 0.48 | 8 (1.8) | 8 (1.7) | 0.95 |
| Coronary heart disease | 3 (3.7) | 0 | 0.11 | 4 (0.9) | 9 (1.9) | 0.18 |
| Atrial fibrillation | 0 | 0 | NA | 2 (0.4) | 2 (0.4) | 1.00 |
| OTT, mean (SD), h | 24.4 (13.0) | 24.3 (13.1) | 0.98 | 24.7 (13.5) | 25.5 (13.8) | 0.37 |
| Time to hospital discharge, median (IQR), d | 11 (9-12) | 11 (10-12) | 0.20 | 11 (10-12) | 11 (10-12) | 0.27 |
| Blood pressure at randomization, mean (SD), mmHg | | | | | | |
| Systolic | 126.6 (8.3) | 128.8 (8.4) | 0.08 | 159.2 (14.8) | 159.7 (14.7) | 0.61 |
| Diastolic | 80.8 (9.5) | 81.7 (11.0) | 0.58 | 91.2 (10.4) | 91.3 (10.1) | 0.92 |
| Mean arterial pressure | 96.1 (7.9) | 97.4 (8.8) | 0.29 | 113.9 (10.4) | 114.1 (9.7) | 0.75 |
| Blood glucose, median (IQR), mmol/L | 6.2 (5.4-7.6) | 6.8 (5.5-9.0) | 0.16 | 6.4 (5.5-8.9) | 6.5 (5.5-8.7) | 0.97 |
| NIHSS score at randomization, median (IQR) | 7 (6-9) | 7 (6-9) | 0.71 | 7 (6-9) | 7 (6-9) | 0.12 |
| Estimated premorbid function (mRS), No. (%) \|\| | | | | | | |
| No symptoms (score, 0) | 54 (65.9) | 64 (72.7) | 0.33 | 326 (72.6) | 330 (71.1) | 0.62 |
| Symptoms without any disability (score, 1) | 28 (34.1) | 24 (27.3) |  | 123 (27.4) | 134 (28.9) |  |
| Presumed stroke cause, No. (%) ¶ | | | | | | |
| Large artery atherosclerosis | 20 (24.4) | 22 (25.0) | 0.27 | 117 (26.1) | 156 (33.8) | 0.08 |
| Small artery occlusion | 1 (1.2) | 1 (1.1) |  | 5 (1.1) | 2 (0.4) |  |
| Cardioembolic | 8 (9.8) | 17 (19.3) |  | 72 (16.1) | 75 (16.2) |  |
| Other determined cause | 2 (2.4) | 0 |  | 6 (1.3) | 4 (0.9) |  |
| Undetermined cause | 51 (62.2) | 48 (54.5) |  | 248 (55.4) | 225 (48.7) |  |

Abbreviations: IQR, interquartile range; SD, Standard Deviation; OTT, time from onset of symptom to remote ischemic conditioning treatment; NIHSS, National Institute of Health Stroke Scale; mRS, modified Rankin Scale.

**Table S12. Baseline Characteristics of Previously diagnosed hypertension patients stratified by SBP 140 mmHg.**

|  | **SBP＜140 mmHg (N=170)** | **SBP≥140 mmHg (N=913)** | ***P* Value** |
| --- | --- | --- | --- |
| Age, mean (SD), y | 65.5 (10.5) | 65.4(10.1) | 0.89 |
| Sex (F), No. (%) | 60 (35.3) | 362 (39.6) | 0.29 |
| Body mass index, median (IQR) | 24.0 (22.5-26.0) | 24.5 (22.9-26.4) | 0.03 |
| Current smoker, No. (%) | 50/163 (30.7) | 230/887 (25.9) | 0.21 |
| Current drinker, No. (%) * | 24/166 (14.5) | 109/892 (12.2) | 0.42 |
| Comorbidities, No. (%) † | | | |
| Hyperlipidemia | 6/165 (3.6) | 14/895 (1.6) | 0.07 |
| Diabetes | 51 (30.0) | 271/911 (29.7) | 0.95 |
| Previous ischemic or hemorrhagic stroke | 76/169 (45.0) | 334/907 (36.8) | 0.05 |
| Previous transient ischemic attack | 1 (0.6) | 16/909 (1.8) | 0.26 |
| Coronary heart disease | 3/169 (1.8) | 13/911 (1.4) | 0.73 |
| Atrial fibrillation | 0 | 4/912 (0.4) | 1.00 |
| OTT, mean (SD), h | 24.3 (13.0) | 25.1(13.7) | 0.37 |
| Time to hospital discharge, median (IQR), d | 10 (9-12) | 11 (10-12) | 0.08 |
| Blood pressure at randomization, mean (SD), mmHg | | | |
| Systolic | 127.7 (8.4) | 159.5 (14.7) | ＜0.001 |
| Diastolic | 81.3 (10.3) | 91.3 (10.2) | ＜0.001 |
| Mean arterial pressure | 96.8 (8.4) | 114 (9.9) | ＜0.001 |
| Blood glucose, median (IQR), mmol/L | 6.3 (5.4-8.2) | 6.5 (5.5-8.8) | 0.33 |
| NIHSS score at randomization, median (IQR) | 7 (6-9) | 7 (6-9) | 0.80 |
| Estimated premorbid function (mRS), No. (%) \|\| | | | |
| No symptoms (score, 0) | 118 (69.4) | 656 (71.9) | 0.52 |
| Symptoms without any disability (score, 1) | 52 (30.6) | 257 (28.1) |  |
| Presumed stroke cause, No. (%) ¶ | | | |
| Large artery atherosclerosis | 42 (24.7) | 273/910 (30.0) | 0.57 |
| Small artery occlusion | 2 (1.2) | 7/910 (0.8) |  |
| Cardioembolic | 25 (14.7) | 147/910 (16.2) |  |
| Other determined cause | 2 (1.2) | 10/910 (1.1) |  |
| Undetermined cause | 99 (58.2) | 473/910 (52.0) |  |

Abbreviations: IQR, interquartile range; SD, Standard Deviation; OTT, time from onset of symptom to remote ischemic conditioning treatment; NIHSS, National Institute of Health Stroke Scale; mRS, modified Rankin Scale.
